# Phase 1 Randomized Study of a Tetravalent Dengue Purified Inactivated Vaccine in Healthy Adults in the United States

**DOI:** 10.4269/ajtmh.16-0634

**Published:** 2017-06-07

**Authors:** Alexander C. Schmidt, Leyi Lin, Luis J. Martinez, Richard C. Ruck, Kenneth H. Eckels, Alix Collard, Rafael De La Barrera, Kristopher M. Paolino, Jean-François Toussaint, Edith Lepine, Bruce L. Innis, Richard G. Jarman, Stephen J. Thomas

**Affiliations:** 1 GSK Vaccines, Rixensart, Belgium; 2 Walter Reed Army Institute of Research, Silver Spring, Maryland; 3 GSK Vaccines, King of Prussia, Pennsylvania

## Abstract

The safety and immunogenicity of four formulations of an investigational tetravalent dengue purified inactivated vaccine (DPIV), formulated at 1 or 4 μg with aluminum hydroxide (alum) or at 1 μg with an adjuvant system (AS01_E_ or AS03_B_), were evaluated in a first-time-in-human, placebo-controlled, randomized, observer-blind, phase 1 trial in the continental United States. Two doses of vaccine or placebo were administered intramuscularly 4 weeks apart to 100 healthy adults 18–39 years of age, randomized 1:1:1:1:1 to receive one of four DPIV formulations or saline placebo. The response to a third dose was evaluated in a subset of nine participants remote from primary vaccination. Humoral immunogenicity was assessed using a 50% microneutralization assay. All DPIV formulations were well tolerated. No vaccine-related serious adverse events were observed through 12 months after the second vaccine dose. In all DPIV groups, geometric mean antibody titers peaked at Day 56, waned through 6 months after the second vaccine dose, and then stabilized. In the nine subjects where boosting was evaluated, a strong anamnestic response was observed. These results support continuation of the clinical development of this dengue vaccine candidate (clinicaltrials.gov: NCT01666652).

## Introduction

Dengue is a mosquito-borne disease caused by any of the four types of serologically distinct, yet antigenically related flaviviruses (dengue virus [DENV]-1, 2, 3, and 4). It is estimated that 390 million dengue infections occur every year, of which 96 million are clinically apparent.^1^,^2^ Several vaccine candidates are at various stages of clinical development.^3^–^5^ Results from three efficacy trials of the Chimerivax (Dengvaxia^™^, Lyon, France; Sanofi Pasteur) live attenuated vaccine have already been published^6^–^8^; and the vaccine has recently received marketing authorization in a number of dengue-endemic countries for use in individuals 9–45 years of age.

The Walter Reed Army Institute of Research (WRAIR), GlaxoSmithKline (GSK) Vaccines, and Fiocruz are pursuing development of a tetravalent dengue purified inactivated vaccine (DPIV) candidate. This approach was encouraged by the safety and efficacy of similar inactivated whole virus vaccines against other flaviviruses, such as the Japanese encephalitis virus (JEV) vaccine Ixiaro^™^ (Valneva Austria GmbH, Vienna, Austria) and the tick-borne encephalitis virus (TBEV) vaccines FSME-IMMUN^™^/TicoVac^™^ (Pfizer Inc., Austria GmbH, Donau, Austria) and Encepur^™^ (GSK Vaccines, Rixensart, Belgium).^9^

Evaluation of adjuvanted DPIV candidates in nonhuman primates identified several DPIV formulations that were highly immunogenic, even at a relatively low antigen dose.^10^ Two doses of DPIV administered 4 weeks apart protected nonhuman primates from viremia (as measured in Vero cell infectivity assays) following challenge with live, near wild-type DENV-1 and DENV-2 administered 8 months and 10 months post-dose 2, respectively. However, antibody titers rose and viral RNA was detectable indicating that immunity in nonhuman primates was not sterile at 8 and 10 months postvaccination.^10^

The WRAIR conducted a proof of concept phase 1 human trial in a small set of healthy, flavivirus-naïve volunteers with monovalent alum-adjuvanted DENV-1 DPIV (2.5 or 5 μg). The safety profile was acceptable and the immunogenicity was low to moderate but supported moving forward with tetravalent DPIV preparations.^11^

We report a first-time-in-human (FTiH) placebo-controlled clinical trial assessing the safety and immunogenicity of four different DPIV formulations at two antigen dose levels, adjuvanted with either aluminum hydroxide (alum) or GSK's adjuvant systems AS01_E_ or AS03_B_, administered intramuscularly on Day 0 and Day 28.

The primary objectives of this study were 1) to evaluate the safety and reactogenicity of four DPIV formulations from Day 0 through Day 28 after the second dose (D56), and 2) to evaluate the humoral immunogenicity to each DENV serotype 28 days after the second dose (D56). The secondary objectives were 1) to evaluate the safety of the four DPIV formulations from Day 0 through 12 months after the second dose (M13) and 2) to evaluate the humoral immunogenicity to each DENV serotype up to M13. In the booster phase, the primary objective was to evaluate the safety, reactogenicity, and immunogenicity of a booster dose administered remote from the primary vaccination series.

The safety, reactogenicity, and humoral immunogenicity of the four DPIV formulations are reported up to 1 year after the second vaccine dose. A small subset of subjects received a booster dose adjuvanted with alum or AS01_E_ in the second year after vaccination, and the safety and immunogenicity of the booster dose are included here.

## Materials and Methods

### Study design.

This study was a phase 1, randomized (1:1:1:1:1), placebo-controlled, observer-blind primary vaccination trial with five parallel groups designed to evaluate the safety and immunogenicity of four DPIV formulations, administered as two doses 4 weeks apart (clinicaltrials.gov: NCT01666652). The study was conducted at the Clinical Trials Center (CTC) at the WRAIR, Silver Spring, MD, in accordance with the Declaration of Helsinki, International Conference on Harmonization, Good Clinical Practice Belmont Principles, and other applicable regulatory and Department of Defense requirements. The protocol and associated documents were reviewed and approved by the WRAIR Institutional Review Board, the Office of Research Protections, Human Research Protection Office, the U.S. Army Medical Materiel Development Activity (USAMMDA), and GSK Vaccines. Internal audits by separate teams from the U.S. Army and GSK Vaccines were also conducted. All participants provided written informed consent prior to study entry.

### Role of the sponsor and development partners.

This study was sponsored by the Surgeon General, Department of the Army. The study design and the protocol were developed collaboratively by WRAIR and GSK study teams. The study was cofunded by the USAMMDA, Military Infectious Diseases Research Program, and GSK Biologicals SA. The USAMMDA, as the Office of the Surgeon General's sponsor representative, monitored and reported on subject safety. The study was conducted under an Investigational New Drug program. Data management and statistical analysis were performed at GSK, according to a prespecified and mutually approved statistical analysis plan. A blinded Safety Review Team and an unblinded Safety Review Committee reviewed safety data at scheduled intervals. All the authors reviewed the manuscript and vouch for its accuracy and completeness.

### Vaccines.

The preparation of the DPIV antigens has been described previously.^10^–^13^ Briefly, DPIV consists of a tetravalent formulation of the following nonattenuated DENV strains: West Pac 74 (DENV-1), S16803 (DENV-2), CH53489 (DENV-3), and TVP360 (DENV-4), propagated in Vero cells, purified, and inactivated with formalin. Clinical lots were prepared following current good manufacturing practices at the WRAIR Pilot Bioproduction Facility, Silver Spring, MD.

Adjuvants used were alum (Alhydrogel 2%, Brenntag Biosector, Frederikssund, Denmark; 10.38 mg of Al^3+^/mL; after dilution, each 0.5-mL vaccine dose contains 500 μg of Al^3+^), and the GSK proprietary adjuvant systems AS01_E_ and AS03_B_. AS01_E_ is an adjuvant system containing 3-*O*-desacyl-4′-monophosphoryl lipid A (MPL; produced by GSK), QS-21 (*Quillaja saponaria* Molina, fraction 21) (licensed by GSK from Antigenics Inc., a wholly owned subsidiary of Agenus Inc., a Delaware, U.S. corporation), and liposome (25 μg MPL and 25 μg QS-21). AS03_B_ is an adjuvant system containing α-tocopherol and squalene in an o/w emulsion (5.93 mg tocopherol).

Four different formulations of the DPIV were used: 1 μg/serotype/dose adjuvanted with alum (1 μg + alum group), AS01_E_ (1 μg + AS01_E_ group) or AS03_B_ (1 μg + AS03_B_ group), and 4 μg/serotype/dose adjuvanted with alum (4 μg + alum group).

The formulations to be adjuvanted with AS01_E_ and AS03_B_ consisted of inactivated vaccine, vialed and freeze-dried. Each vial, corresponding to one dose, contained 1 μg of each DENV serotype. DPIV was reconstituted at the time of administration by mixing the freeze-dried product with the appropriate adjuvant system. To prepare DPIV with alum, monovalent bulk vaccine lots were first diluted in buffer and combined to create the tetravalent formulation at either 1 μg/serotype/dose or 4 μg/serotype/dose. The formulated, tetravalent bulk was adsorbed on alum for 1 hour, and then vialed and stored at 2–8°C.

A solution of 154 mM sodium chloride was used as placebo. Placebo and vaccine injection volumes were identical (0.5 mL).

All DPIV formulations and placebos were administered intramuscularly in the deltoid muscle at study days 0 and 28. The booster recipients received a third dose of the same vaccine formulation they received during primary vaccination (1 μg + AS01_E_ or 4 μg + alum).

### Study participants.

Healthy male and female adults between 18 and 39 years of age were recruited by staff from the WRAIR CTC. Volunteers were provided with a detailed explanation of the study and enrolled after an informed consent process. Female participants had to be of nonchildbearing potential or abstinent, or had to use adequate contraceptive precautions for 30 days prior to vaccination, had a negative pregnancy test on the day of vaccination, and agreed to continue such precautions for 60 days after completion of the vaccination series. Volunteers initially seropositive for hepatitis B surface antigen, hepatitis C virus antibodies, or human immunodeficiency virus antibodies were excluded. Other exclusion criteria were a history of chronic disease, chronic alcohol consumption and/or drug abuse, receipt of immunoglobulins and/or any blood products within 90 days preceding vaccination or planned administration during the study period, as well as clinically significant laboratory abnormalities.

Participants were not screened for flavivirus antibodies at enrollment but the according-to-protocol (ATP) analysis of immunogenicity included only participants who were seronegative for all four DENV serotypes at baseline (microneutralization assay [MN50] titer < 1:10). Serostatus for DENV was determined after enrollment based on prevaccination blood samples.

In total, 100 participants were enrolled and randomized 1:1:1:1:1 to receive one of the four DPIV formulations or saline placebo. The randomization was performed using MATEX, a program developed for use in SAS (SAS Institute Inc., Cary, NC). Treatment allocation at the investigator site was performed using a central randomization system on internet. The experimental vaccination numbers were allocated by dose (dose 1 or dose 2). The randomization algorithm used a minimization procedure accounting for center to determine the experimental vaccination box number to be used for the subject.

In the booster phase (15–21 months after the second vaccine dose), eligible participants were healthy adults, who had completed the primary study and received two doses of either the 1 μg + AS01_E_ or the 4 μg + alum vaccine formulation in the primary phase. Randomly ordered listings of subjects in those two groups were used to target enrollment of five subjects per group—with the possibility to have subjects from the other group added in case less than five subjects consented to participate in one group. Additionally, participants were excluded from the booster phase if they had received any vaccination within 4 weeks prior to booster vaccination.

A small subset of participants (*N* = 9) from the 4 μg + alum (*N* = 3) and 1 μg + AS01_E_ (*N* = 6) were enrolled in the booster phase after an informed consent process and vaccinated with the same formulation as the primary doses.

### Blinding.

The study was observer-blind, with the vaccine/placebo recipients and those responsible for the evaluation of all study endpoints being unaware of treatment assignments. Vaccine preparation was performed at the WRAIR Pilot Bioproduction Facility and administration of the vaccine was done at the WRAIR CTC by nurse coordinators who did not participate in any of the clinical study evaluation activities. The laboratories in charge of testing were blinded with regard to treatment allocation, and codes were used to link the subject and study to each sample.

### Safety evaluation.

Solicited injection site and general adverse events (AEs) were recorded on diary cards for 7 days after each dose. Solicited injection site AEs included pain, redness, and swelling. Solicited general AEs included fatigue, fever, headache, gastrointestinal symptoms (nausea, vomiting, diarrhea, and/or abdominal pain), joint pain, and muscle ache. Intensities of each AE were scored as grades 1–3, with grade 3 fever defined as an oral body temperature ≥ 39°C, grade 3 redness and swelling defined as ≥ 101-mm diameter around the injection site, and all other grade 3 AEs defined as those events preventing normal daily activity.

Unsolicited AEs were recorded for 28 days after each dose, and were coded with the use of the Medical Dictionary for Regulatory Activities (MedDRA).^14^ Serious adverse events (SAEs), potential immune-mediated diseases (pIMDs), and medically attended events (MAEs) were recorded throughout the entire study period. SAEs were defined as medically significant events, including those AEs resulting in hospitalization, disability, or death. pIMDs are a subset of AEs that include autoimmune diseases and other inflammatory or neurologic disorders of interest which may or may not have an autoimmune etiology. An MAE is an AE for which the subject received medical attention defined as hospitalization, an emergency room visit, or a visit to or from medical personnel.

Safety laboratory assessments, including a complete blood count (red blood cell count, white blood cell count [WBC], hemoglobin [Hgb], hematocrit, platelet count, differential and a calculated absolute neutrophil count), alanine aminotransferase (ALT), and aspartate aminotransferase (AST) levels, blood urea nitrogen, creatinine, alkaline phosphatase, and total and direct bilirubin, were performed prior to, 7 and 28 days after each vaccination, and 3 (M4), 6 (M7), 9 (M10), and 12 (M13) months after the second dose. In the booster phase, the safety laboratory assessments were not repeated at screening if they were performed and found to be within acceptable ranges within 90 days of the planned booster vaccination. All safety-related clinical laboratory values were reviewed and all abnormal values were assessed by the investigators as clinically significant or not, with respect to safety.

Because this was an FTiH study, several additional safety measures were incorporated into the study design. Enrollment occurred in two waves, starting with enrollment and evaluation of the safety data collected up to 7 days postvaccination for 20 participants (four participants per group), prior to enrollment and administration of study vaccine to Wave 2 subjects. A Safety Review Committee reviewed unblinded safety data at three scheduled intervals. The first scheduled review took place once the 20 participants from Wave 1 had completed the 7 days of safety follow-up post-dose 1 (Days 0–6) and included all safety data available up to this time point. After the first scheduled Safety Review Committee review resulted in a recommendation to proceed and the sponsor approved, the second dose was administered to participants from Wave 1, and participants from Wave 2 were enrolled and vaccinated. The second scheduled review took place once the 20 participants in Wave 1 had completed the 7 days of safety follow-up post-dose 2 and included all available safety data from both waves. Dose 2 administration to Wave 2 participants occurred after the second unblinded safety report was reviewed by the Safety Review Committee, resulted in a recommendation to proceed, and was approved by the sponsor. The third scheduled review took place once 60 volunteers were enrolled, and Day 7 post-dose 1 safety data were available for all 60 volunteers.

### Immunogenicity assessment.

Blood samples were collected on the day of each vaccination and 28 days after each dose, and 3, 6, and 12 months after the second dose, that is, M4, M7, and M13 in the primary vaccination phase; prevaccination, 7, and 28 days postvaccination in the booster phase. To characterize DPIV immunogenicity, anti-DENV neutralizing antibodies (Nab) were determined for the above-mentioned time points. Antibodies to each DENV serotype were measured at the Pilot Bioproduction Facility, WRAIR, using a 96-well quantitative MN50 performed in Vero cells as previously described.^15^,^16^ Seropositivity was defined as a titer ≥ 1:10. Peripheral blood mononuclear cells were collected for B- and T-cell assays. These assessments are ongoing and will be published at a later time.

Antibody avidity, a measure of antibody quality described previously,^10^ was also determined for pre- and postvaccination sera. Briefly, 50 μL/well of DENV antigen (sucrose gradient-purified live virus) were coated onto 96-well high-binding enzyme-linked immunosorbent assay (ELISA) plates (Costar 2592) and stored overnight at 2–8°C. After incubation, the plates were blocked with 300 μL/well of antibody-blocking diluent (ABD), consisting of nonfat dry milk in 1× phosphate-buffered saline (PBS) for 30 minutes at 20–25°C. Heat-inactivated test serum samples diluted 1:100 in ABD were added to quadruplicate wells of DENV antigen-coated wells (50 μL/well). After 2 hours at 35–37°C incubation, the wells were aspirated and half of the wells were incubated for 10 minutes at 20–25°C with 200 μL/well of 1× PBS, whereas the other half of the wells were incubated with 200 μL/well of 8 M urea. After a 10-minute incubation, bound IgG was detected by washing all the plates five times with 1× PBS and adding a qualified dilution of goat-anti-human IgG-horse radish peroxidase (HRP 04-10-06, KPL, Gaithersburg, MD) in ABD (50 μL/well) to all the wells. After 1 hour at 35–37°C, the plates were washed five times with 1× PBS and bound antibodies were visualized by adding 50 μL/well of tetramethylbenzidine. After 8–10 minutes of blue color development, the reaction was stopped by adding 50 μL/well of a 1:25 dilution of phosphoric acid. The plates were read immediately at a wavelength of 450 nm. The optical density (OD) values for each serum sample were normalized by subtracting OD values obtained using the ELISA blanks (negative-normal human sera) for each 96-well plate. The avidity index (AI) was expressed as the ratio of the OD of wells treated with urea to the OD of wells treated without urea (1 × PBS) and converted to a percentage value.

### Statistical analysis.

This study was exploratory; thus, all analyses were descriptive, with no statistical comparisons performed. The analysis of safety and reactogenicity in the primary phase was performed on the total vaccinated cohort (TVC; i.e., all participants who received at least one vaccine dose), and the immunogenicity analysis in the primary phase was based on the ATP cohorts for immunogenicity at D56, M7, and M13 (initially seronegative participants who met all eligibility criteria, complied with protocol procedures, had no elimination criteria during the study, and had immunogenicity data available at D56, M4, M7, and M13). In the booster phase, safety analysis was performed on the booster TVC (all participants who received the booster dose) and the immunogenicity analysis was based on the booster ATP cohort for immunogenicity, which included all booster-vaccinated participants (i.e., those who met all eligibility criteria, complied with the procedures defined in the protocol, and had no elimination criteria) for whom data concerning immunogenicity endpoint measures were available prebooster and 28 days postbooster.

The percentage of doses followed by each individual solicited injection site and general AE were recorded by group with exact 95% confidence intervals (CIs). All SAEs occurring during the study were listed for each treatment group. All SAEs, pIMDs, pregnancies, and all related to treatment AEs were described in detail. Grade 3 or higher laboratory hematological and biochemical parameters were described in detail.

The following immunogenicity parameters were calculated by group, each with exact 95% CIs: monovalent, bivalent, trivalent, and tetravalent seropositivity rates; and geometric mean titers (GMTs) for each DENV serotype. Monovalent, bivalent, trivalent, and tetravalent seropositivity rates were defined as the percentage of participants who were seropositive to 1, 2, 3, and all 4 DENV serotypes, respectively. The distribution of antibody titers for each DENV serotype was summarized by reverse cumulative curves.

## Results

### Study population.

**Figure 1.**
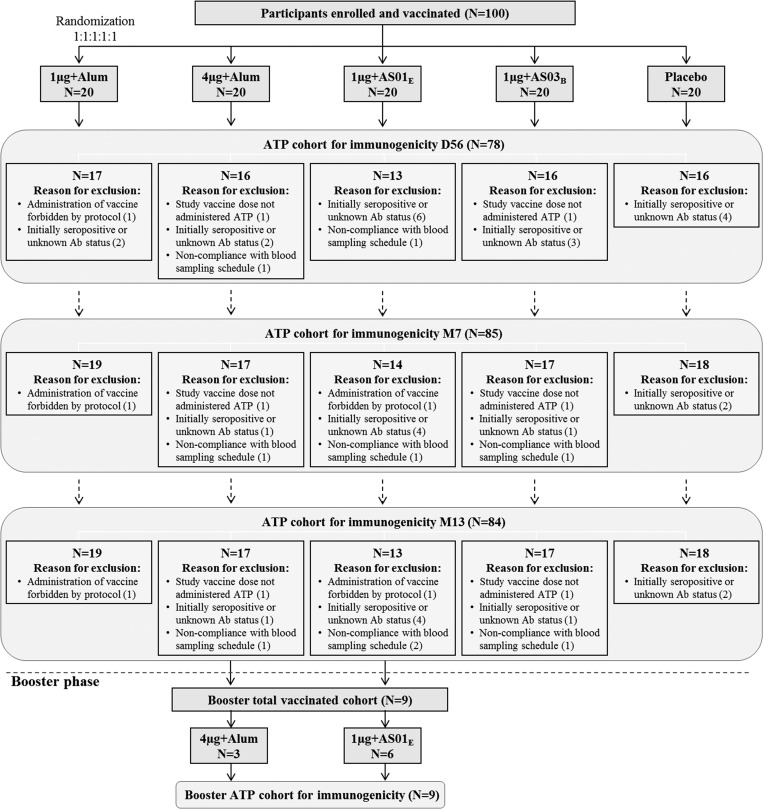
Disposition of study participants and reasons for exclusion from according-to-protocol cohort for immunogenicity. 1 μg + alum indicates participants who received 1 μg/serotype/dose adjuvanted with alum; 4 μg + alum indicates participants who received 4 μg/serotype/dose adjuvanted with alum; 1 μg + AS01_E_ indicates participants who received 1 μg/serotype/dose adjuvanted with AS01_E_; 1 μg + AS03_B_ indicates participants who received 1 μg/serotype/dose adjuvanted with AS03_B_. *N* = number of participants; ATP = according-to-protocol; Ab = antibody. D56 = Day 56, (1 month post-dose 2); M7 = Month 7 (6 months post-dose 2); M13 = Month 13 (12 months post-dose 2). Dashed arrows were used between the different ATP cohorts for immunogenicity to show chronological order (D56, M7, M13), but a cohort is not embedded in the preceding one.

A total of 100 participants (20 per group) were enrolled from August 2012 to March 2013, received at least one dose of vaccine or placebo, were included in the TVC, and were followed for 13 months; 98 participants received two doses and a total of 91 participants completed the study through M13. Twenty-two, 15, and 16 participants (of 100) were excluded from the ATP cohorts for immunogenicity D56, M7, and M13, respectively. The reasons for exclusion are listed in Figure 1

.

The mean age in the TVC at first vaccination was 27.8 years (median = 27 years; range = 18–39 years); 43% of participants were female; 51% self-identified as African American; 34% as Caucasian (Table 1).

Two pregnancies were reported; one in the 1 μg + AS03_B_ group, last menstrual cycle 8 months post-dose 2, resulting in a spontaneous abortion at 8 weeks of gestation and one in the placebo group, last menstrual cycle 3.5 months post-dose 2, resulting in the birth of a healthy baby at 36 weeks of gestation.

In the booster phase of the study, one dose of DPIV was administered to nine participants (booster TVC) from the 4 μg + alum group (three participants) and the 1 μg + AS01_E_ group (six participants). All nine participants were included in the booster ATP immunogenicity cohort. The mean age in the booster TVC at first vaccination was 27.8 years (median = 26 years; range = 21–37 years); 33% of participants were female; 67% were African American and 33% Caucasian.

### Reactogenicity and safety.

**Figure 2.**
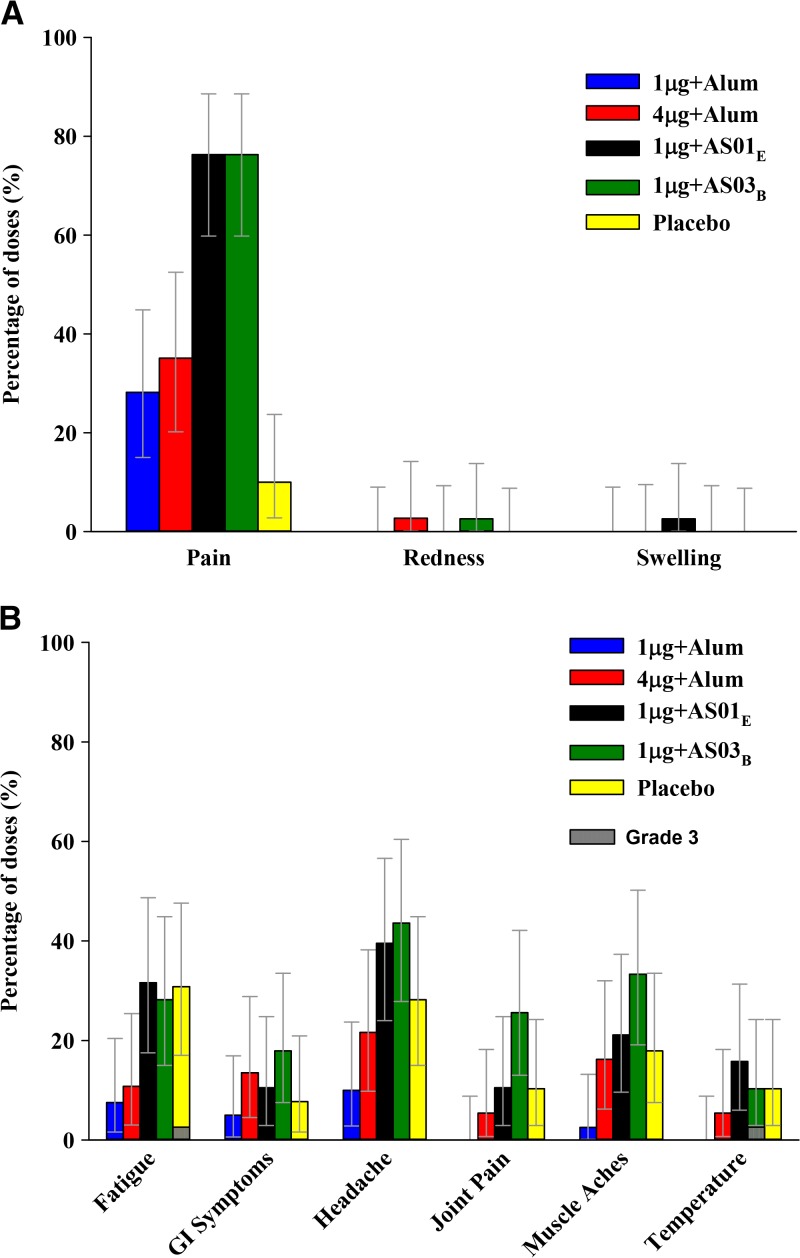
Overall per dose incidence of any grade solicited injection site (**A**) and general (**B**) adverse events during the 7-day postvaccination period (total vaccinated cohort). 1 μg + alum indicates participants who received 1 μg/serotype/dose adjuvanted with alum; 4 μg + alum indicates participants who received 4 μg/serotype/dose adjuvanted with alum; 1 μg + AS01_E_ indicates participants who received 1 μg/serotype/dose adjuvanted with AS01_E_; 1 μg + AS03_B_ indicates participants who received 1 μg/serotype/dose adjuvanted with AS03_B_. Error bars indicate exact 95% confidence intervals; GI = gastrointestinal; Temperature = oral temperature ≥ 37.5°C. Intensities of each AE were scored as grades 1–3, with grade 3 fever defined as an oral body temperature ≥ 39°C, grade 3 redness and swelling defined as ≥ 101-mm diameter around the injection site, and all other grade 3 AEs defined as those events preventing normal daily activity.

In the primary vaccination phase, most subjects (90–100% per group) returned a symptom diary card after vaccination. All reported solicited injection site AEs were of mild or moderate intensity. Injection site pain was more frequently reported by participants receiving AS01_E_- or AS03_B_-adjuvanted vaccine than by those receiving alum-adjuvanted vaccine or placebo (Figure 2A

). Solicited general AEs were mostly of mild or moderate intensity and occurred with similar frequencies in all treatment groups (Figure 2B). Two grade 3 general solicited AEs were reported: one grade 3 fever (39.4°C on Day 2 post-dose 2, single measurement, duration 1 day) in the 1 μg + AS03_B_ group considered as possibly related to vaccination, and one grade 3 fatigue on Day 6 post-dose 2 (duration 1 day) in the placebo group considered as not related to vaccination.

In the booster vaccination phase, injection site pain was the most common solicited injection site AE during the 7-day postvaccination period, reported by two of three participants in the 4 μg + alum group (one grade 1 and one grade 2, duration 2 days for each) and four of six participants (three participants reported grade 1 pain, with a mean duration of 2.3 days; one participant experienced a grade 2 worsening for 1 day) in the 1 μg + AS01_E_ group. Redness was reported by one participant from the 4 μg + alum group. Grade 3 injection site AEs were not observed. No solicited general AEs were reported in the 4 μg + alum group. The reported solicited general AEs in the 1 μg + AS01_E_ group were fatigue (three of six participants), headache (one of six), joint pain (one of six), muscle aches (three of six participants), and fever (two of six participants). One case of grade 3 muscle aches was reported on Day 1 in the 1 μg + AS01_E_ group (duration 1 day, considered as possibly related to vaccination).

Concerning hematological and biochemical levels, there were four participants in the primary vaccination phase with a grade 3 or higher laboratory value (which were normal at screening; none were considered related to vaccination): one participant in the 1 μg + AS01_E_ group had a grade ≥ 3 AST elevation of 557 U/L at D35 (ALT was 170 U/L). The participant reported heavy alcohol use in the days prior to the D35 visit and was advised to avoid alcohol. ALT and AST had returned to normal at the D56 visit. One participant in the placebo group had a neutrophil count of 990 per μL at D35. The neutrophil count returned to normal by D56. At M4, one participant in the 1 μg + AS03_B_ group had an AST of 781 U/L (ALT = 220 U/L, creatinine phosphokinase [CPK] > 50,000 U/L) considered due to unusually excessive physical exercise. Within 8 days, AST and ALT returned to normal and CPK dropped to 409 (upper limit of normal [ULN] 196). One subject had an Hgb level of 8.4 g/dL at her M4 visit. She had had elective surgery 9 days before her M4 visit. Hgb value improved subsequently.

In the primary vaccination phase, during the 28-day postvaccination period, unsolicited symptoms were reported by 50%, 45%, 50%, 70%, and 75% of participants in the 1 μg + alum, 4 μg + alum, 1 μg + AS01_E_, 1 μg + AS03_B_, and placebo groups, respectively. No grade 3 vaccine-related unsolicited AEs were reported in any group. There were four grade 3 unsolicited AEs considered not related to vaccination: one upper respiratory tract infection (placebo group), one increased AST (1 μg + AS01_E_ group), one decreased neutrophil count (placebo group), and one headache (1 μg + alum group). At the “preferred term” level, the most frequently reported unsolicited AEs per group (those reported by more than one participant per group) were 1 μg + alum: nasopharyngitis (10%); 1 μg + AS01_E_: chills (10%); 1 μg + AS03_B_: upper respiratory tract infection (15%) and nasopharyngitis (10%); placebo: upper respiratory tract infection (15%), decreased neutrophil count (15%), nasopharyngitis (10%), bradycardia (10%), tachycardia (10%), and increased WBC count (10%); no AE was reported by more than one participant in the 4 μg + alum group. Of note, the study was conducted during the fall and winter months when increased respiratory tract signs and symptoms are anticipated.

Four SAEs were observed through M13 in the primary vaccination phase: three in the 1 μg + AS03_B_ group (abdominal discomfort, death due to trauma [gunshot wound], and spontaneous abortion, reported at 49, 226, and 298 days post-dose 2, respectively) and one in the 1 μg + AS01_E_ group (asthma exacerbation, 360 days post-dose 2); these SAEs were assessed by the investigator as not related to vaccination. MAEs were reported by one subject (bacterial vaginosis) in the 1 μg + alum group, one subject (sinusitis) in the 4 μg + alum group, two subjects (back pain and asthma) in the 1 μg + AS01_E_ group, five subjects (abdominal discomfort, influenza like illness, upper respiratory tract infection, gunshot wound, and spontaneous abortion) in the 1 μg + AS03_B_ group, and two subjects (ear infection, streptococcal pharyngitis, and upper respiratory tract infection) in the placebo group, up to M13. No pIMDs were reported through M13.

In the booster vaccination phase, no participant reported unsolicited symptoms, grade 3 or higher laboratory values, SAEs, pIMDs, or pregnancies up to 28 days after booster vaccination.

### Humoral immunogenicity.

**Figure 3.**
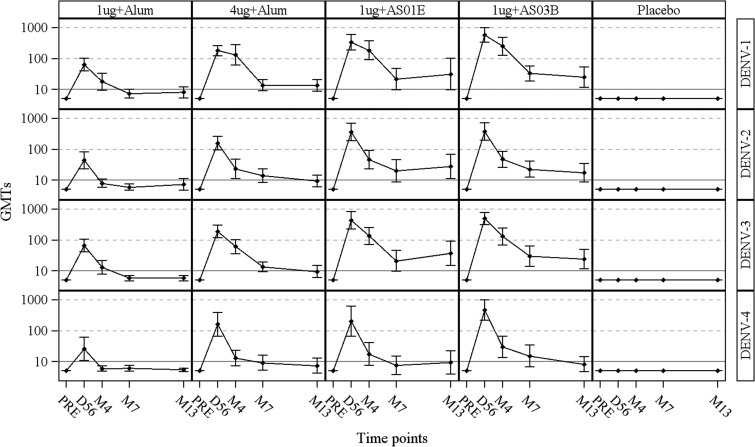
Geometric mean titers to dengue virus types 1–4 up to 1 year post-dose 2 (according-to-protocol cohort for immunogenicity M13). 1 μg + alum indicates participants who received 1 μg/serotype/dose adjuvanted with alum; 4 μg + alum indicates participants who received 4 μg/serotype/dose adjuvanted with alum; 1 μg + AS01_E_ indicates participants who received 1 μg/serotype/dose adjuvanted with AS01_E_; 1 μg + AS03_B_ indicates participants who received 1 μg/serotype/dose adjuvanted with AS03_B_. Participants with a titer below the assay cutoff of 10 were attributed a titer of 5; DENV = dengue virus type; PRE = prevaccination; D56 = Day 56 (1 month post-dose 2); M4 = Month 4 (3 months post-dose 2); M7 = Month 7 (6 months post-dose 2); M13 = Month 13 (12 months post-dose 2). Error bars indicate 95% confidence intervals.

**Figure 4.**
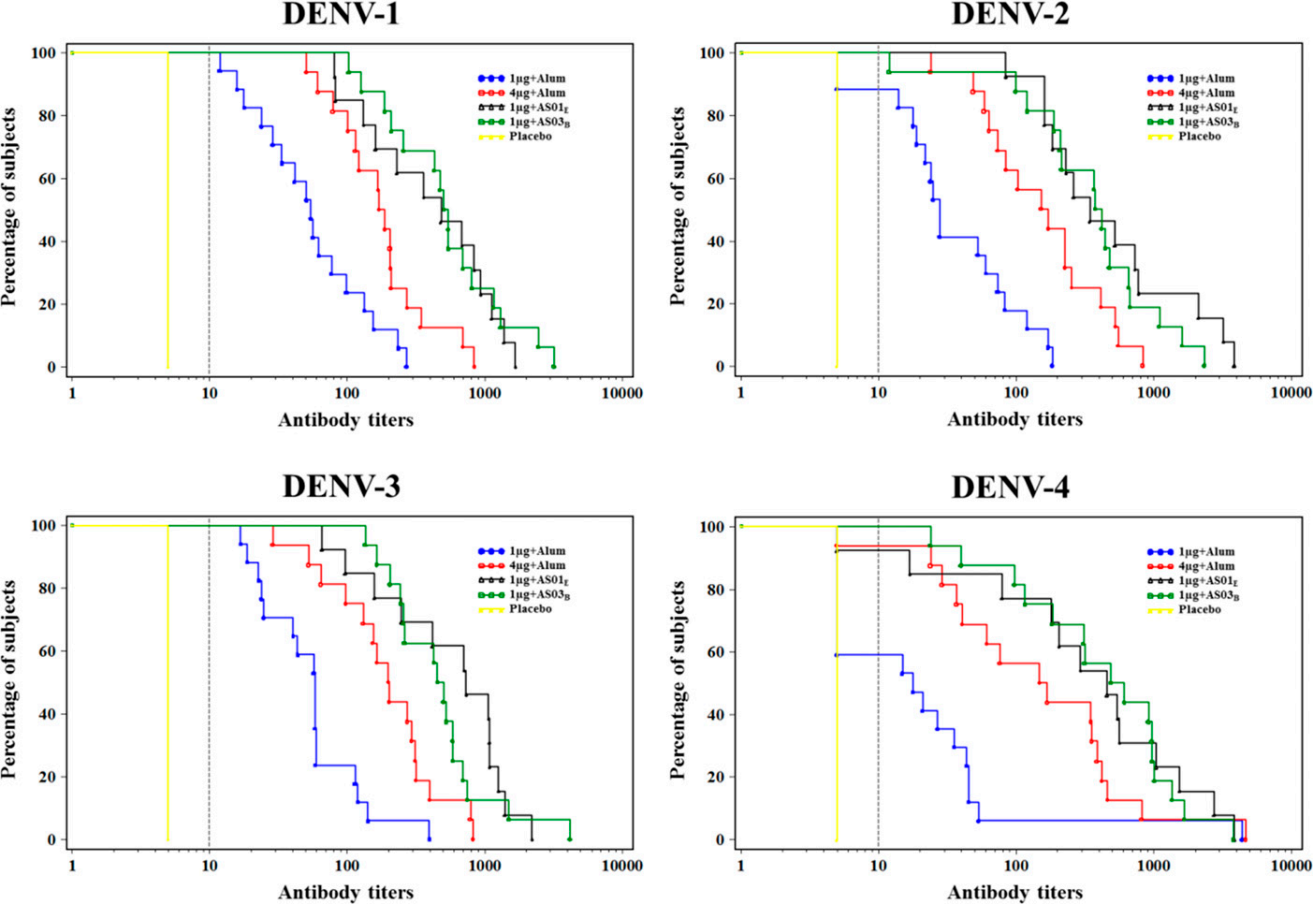
Reverse cumulative distribution curves for antibody titers to dengue virus types 1–4 at D56 (according-to-protocol cohort for immunogenicity D56). 1 μg + alum indicates participants who received 1 μg/serotype/dose adjuvanted with alum; 4 μg + alum indicates participants who received 4 μg/serotype/dose adjuvanted with alum; 1 μg + AS01_E_ indicates participants who received 1 μg/serotype/dose adjuvanted with AS01_E_; 1 μg + AS03_B_ indicates participants who received 1 μg/serotype/dose adjuvanted with AS03_B_. Dotted line indicates cutoff for MN50 = 10 ED_50_; participants with a titer below the assay cutoff were attributed the arbitrary value of half the cutoff. MN50 = 50% microneutralization assay; DENV = dengue virus type.

DENV-Nab GMTs are presented in Figure 3

(ATP cohort for immunogenicity M13) and Table 2 (ATP cohorts for immunogenicity at D56, M7, and M13). At D56, the 1 μg + alum formulation induced GMTs between 20 (DENV-4) and 55 (DENV-1), whereas the 4 μg + alum formulation induced GMTs ~3–7 times higher than 1 μg + alum (from 141 [DENV-4] to 191 [DENV-3]). The AS01_E_- and AS03_B_-adjuvanted formulations induced the highest GMTs. D56 GMTs in the AS01_E_-adjuvanted group were approximately 7- to 15-fold higher than in the 1 μg + alum group, whereas GMTs in the AS03_B_-adjuvanted group were approximately 8- to 20-fold higher than in the 1 μg + alum group (Table 2). GMTs waned from D56 to M7, then stabilized through M13 in all DPIV groups (Figure 3). Reverse cumulative distribution curves for DENV-1, 2, 3, and 4 at the D56 visit are shown in Figure 4

.

**Figure 5.**
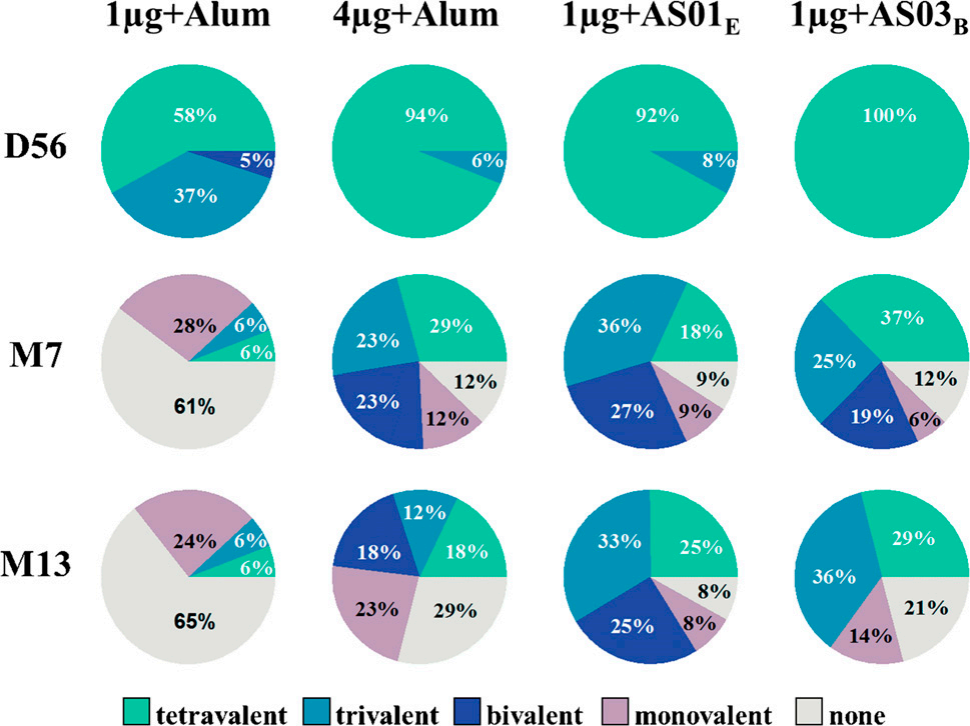
Seroconversion rates to dengue virus types 1–4 up to 1 year post-dose 2 (according-to-protocol cohort for immunogenicity M13). 1 μg + alum indicates participants who received 1 μg/serotype/dose adjuvanted with alum; 4 μg + alum indicates participants who received 4 μg/serotype/dose adjuvanted with alum; 1 μg + AS01_E_ indicates participants who received 1 μg/serotype/dose adjuvanted with AS01_E_; 1 μg + AS03_B_ indicates participants who received 1 μg/serotype/dose adjuvanted with AS03_B_. Monovalent, bivalent, trivalent, and tetravalent seropositivity rates are defined as the percentage of subjects who are seropositive to 1, 2, 3, and all 4 DENV types, respectively. No seroconversion was observed in the placebo group, which was therefore not shown. Not all numbers add up to 100% due to rounding. D56 = Day 56 (1 month post-dose 2); M7 = Month 7 (6 months post-dose 2); M13 = Month 13 (12 months post-dose 2).

Tetravalent DENV antibody seroconversion rates at D56 were 58% in the 1 μg + alum group and 92–100% in the 4 μg + alum, 1 μg + AS01_E_, and 1 μg + AS03_B_ groups. Seropositivity rates decreased over time (Figure 5

), as expected from the waning GMTs (c.f. Table 2), with the lowest M13 seropositivity rates for DENV-4 (5.9% in the 1 μg + alum group to 28.6% in the 1 μg + AS03_B_ group, whereas percentages for DENV-1 to 3 ranged between 11.8% and 83.3%).

**Figure 6.**
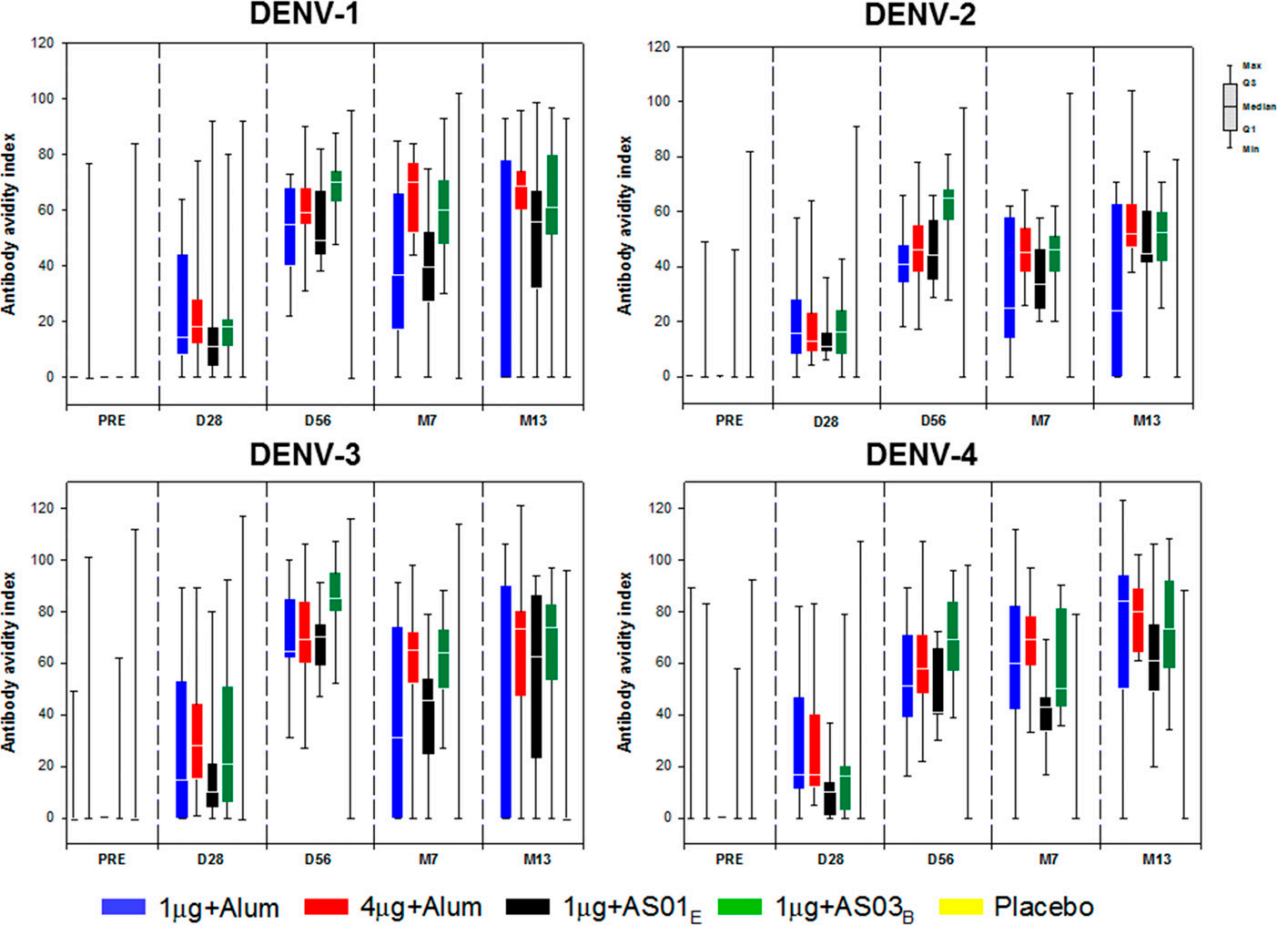
Box and whisker plot of avidity index for dengue virus types 1–4 up to 1 year post-dose 2 (adapted according-to-protocol cohort for immunogenicity). 1 μg + alum indicates participants who received 1 μg/serotype/dose adjuvanted with alum; 4 μg + alum indicates participants who received 4 μg/serotype/dose adjuvanted with alum; 1 μg + AS01_E_ indicates participants who received 1 μg/serotype/dose adjuvanted with AS01_E_; 1 μg + AS03_B_ indicates participants who received 1 μg/serotype/dose adjuvanted with AS03_B_. DENV = dengue virus type; PRE = prevaccination; D28 = Day 28 (1 month post-dose 1); D56 = Day 56 (1 month post-dose 2); M7 = Month 7 (6 months post-dose 2); M13 = Month 13 (12 months post-dose 2). According to the time point, the following cohorts were used to create the adapted according-to-protocol cohort for immunogenicity: for PRE, D28, D56: ATP cohort for immunogenicity D56; for M7: ATP cohort for immunogenicity M7 and for M13: ATP cohort for immunogenicity M13. Antibody avidity indices describe polyclonal antibody binding in the absence or presence of 8 M urea, as described in the Materials and Methods section. Boxes and/or whiskers with limits equal to 0 are flattened to a horizontal bar.

**Figure 7.**
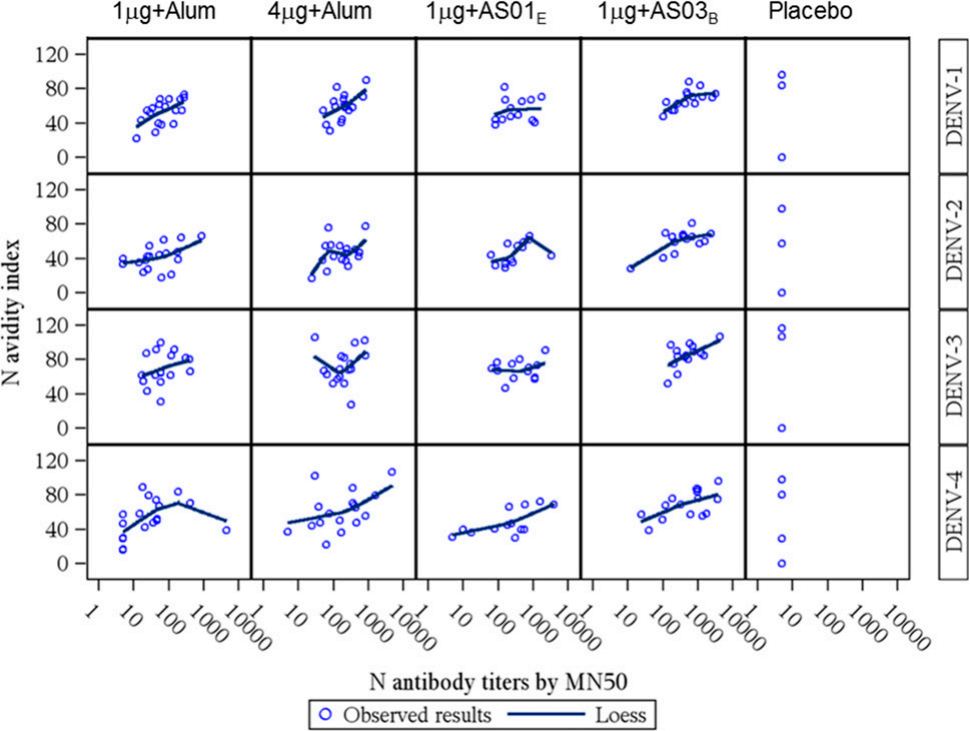
Avidity index vs. the *N* antibody titers by MN50 for dengue virus serotypes 1–4 at D56 (adapted according-to-protocol cohort for immunogenicity). 1 μg + alum indicates participants who received 1 μg/serotype/dose adjuvanted with alum; 4 μg + alum indicates participants who received 4 μg/serotype/dose adjuvanted with alum; 1 μg + AS01_E_ indicates participants who received 1 μg/serotype/dose adjuvanted with AS01_E_; 1 μg + AS03_B_ indicates participants who received 1 μg/serotype/dose adjuvanted with AS03_B_. DENV = dengue virus serotype; D56 = Day 56 (1 month post-dose 2); Loess = nonparametric local regression fit. According to the time point, the following cohorts were used to create the adapted according-to-protocol cohort for immunogenicity D56: for PRE, D28, D56: ATP cohort for immunogenicity; for M7: ATP cohort for immunogenicity M7 and for M13: ATP cohort for immunogenicity M13.

Across all active vaccine treatment groups, medium to high antibody avidity indices were observed from D56 onward (Figure 6

). In post hoc exploratory analyses, a rise in DENV 1–4 avidity indices was observed in all active groups compared with placebo on D56. The difference was still detected in the 4 μg + alum and 1 μg + AS03_B_ groups at M7 and M13. Higher antibody titers were generally associated with higher avidity indices (Figure 7

).

**Figure 8.**
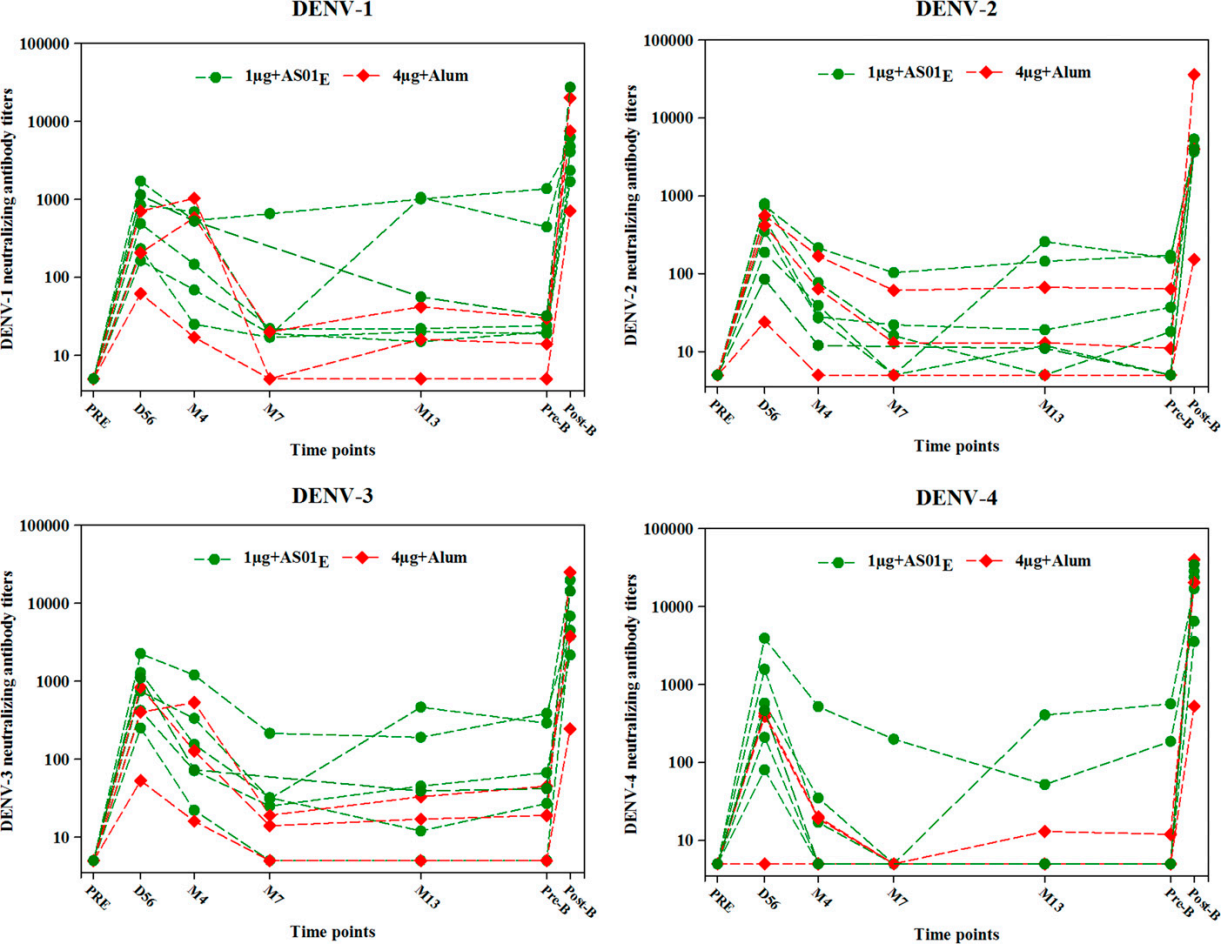
Neutralizing antibody titers to dengue virus serotypes 1–4 up to 28 days postbooster (booster according-to-protocol cohort for immunogenicity). 4 μg + alum indicates participants who received 4 μg/serotype/dose adjuvanted with alum; 1 μg + AS01_E_ indicates participants who received 1 μg/serotype/dose adjuvanted with AS01_E_. Participants with a titer below the assay cutoff were attributed the arbitrary value of half the cutoff; DENV = dengue virus serotype; PRE = prevaccination; D56 = Day 56 (1 month post-dose 2); M7 = Month 7 (6 months post-dose 2); M13 = Month 13 (12 months post-dose 2); Pre-B = prebooster vaccination, Post-B = 28 days postbooster vaccination.

The nine subjects who received a booster dose between 15 and 21 months post-dose 2 responded with a brisk rise in Nab titers to each DENV serotype (Table 3 and Figure 8

). In eight subjects, Nab titers rose from modest or even undetectable titers to titers in the thousands.

## Discussion

In this FTiH phase 1 study of an inactivated DENV vaccine candidate, four different DPIV formulations were found to be well tolerated and immunogenic. Transient mild to moderate injection site pain was reported by the majority of participants receiving formulations that contained non-alum adjuvants (AS03_B_ or AS01_E_) with no grade 3 injection site solicited symptoms observed. Similar findings have been reported for other AS01- and AS03-adjuvanted vaccines.^17^,^18^ Solicited general symptoms occurred with similar frequencies in all treatment groups. One vaccinee in the 1 μg + AS03_B_ group developed grade 3 fever 2 days post-dose 2 that lasted less than 1 day. pIMDs, vaccine-related SAEs, or grade 3 vaccine-related unsolicited AEs were not reported in any group through the M13 visit. The booster dose was also well tolerated, suggesting that both primary and booster doses of various DPIV formulations have a clinically acceptable reactogenicity profile in adults.

Consistent with the results of preclinical studies in nonhuman primates,^10^ AS01_E_- and AS03_B_-adjuvanted DPIV formulations as well as the high-dose alum-adjuvanted DPIV formulation induced a balanced and high-titer Nab response against all four DENV serotypes in dengue-seronegative adults after two intramuscular injections of DPIV 4 weeks apart. The potent antibody response achieved (GMTs in naïve subjects against all four serotypes above 300 with AS01_E_ or AS03_B_ and above 100 with 4 μg + alum), as well as the balanced immunogenicity against all four serotypes (GMTs differed no more than 2-fold among serotypes within a group) were encouraging.

Live attenuated dengue vaccines appear to contain an immunologically dominant strain, that is, one strain appears more replication competent and more immunogenic than the other strains, resulting in Nab titers that differ by up to 20- to 100-fold after the first dose in flavivirus-naïve subjects.^19^–^21^ The kinetics of Nab titers suggests that the first dose of these attenuated vaccines induces robust immunity against the dominant strain, but not necessarily against the other three strains: Nab titers against the dominant serotype are highest post-dose 1, whereas Nab titers against the nondominant strains are boosted by subsequent vaccine doses. It has been suggested that differences in vaccine virus replication competence might lead to differences in immunogenicity and subsequently to differences in DENV serotype-specific clinical efficacy.^6^–^8^,^21^ Avoiding differences in vaccine virus replication competence and/or viral interference was one reason to pursue an inactivated dengue vaccine development program.

In contrast with the results in primed individuals,^22^ Nab GMTs waned considerably from D56 to M7 and then stabilized at various levels depending on the vaccine formulation. The majority of subjects in the low-dose alum group had turned seronegative by M7, and M7 GMTs in the high-dose alum group were close to the limit of detection of 10. Although titers in the AS01 and AS03 groups were higher than in the high-dose alum group, it is uncertain whether two doses of vaccine administered 1 month apart can provide long-term protection from disease for flavivirus-naïve vaccinees. GMTs waned in the preclinical studies as well, but peak titers and plateau titers after M7 were higher in nonhuman primates than in flavivirus-naïve human subjects. Rhesus monkeys were protected against viremia (measured by virus isolation) following wild type DENV challenge at 8 and 10 months post-dose 2, and strong B-cell memory responses were observed.^10^ The waning and relatively low plateau of Nab GMTs observed in the current study are reminiscent of the Nab kinetics seen following two doses of other whole virus inactivated alum-adjuvanted flavivirus vaccines, for example, JEV and TBEV vaccines. Longitudinal studies for these licensed vaccines, containing similar amounts of viral protein (6 μg and 1.5 μg for JEV and TBEV, respectively) adjuvanted with alum show that the decline in GMTs following the primary series is pronounced during the first year and then levels off.^23^,^24^ Nab waning is expected, and the intent of vaccination is to induce sufficient immunologic memory so that adaptive immune responses are robust, swift, and protective when natural infection occurs. A third dose remote from the primary vaccination series may be warranted for individuals who remain at risk for DENV infection.

The primary immunogenicity results (GMTs and tetravalent seroconversion) from our study appear to be superior 1 month postvaccination to those previously reported using live attenuated formulations in flavivirus-unprimed adults (measured with the same MN50 assay).^15^ As a comparison, tetravalent seropositivity rates with WRAIR/GSK tetravalent dengue live attenuated vaccine (LAV) formulations ranged between 60% and 66.7% 1 month post-dose 2 in unprimed recipients.^15^ In our study, tetravalent seropositivity rates at 1 month after the second DPIV dose were 94%, 92%, and 100% in the 4 μg + alum, 1 μg + AS01_E_, and 1 μg + AS03_B_ groups, respectively. Despite the apparent superiority of the DPIV formulations compared with LAV, in terms of peak Nab titers, it is not clear yet whether Nab titers, as currently determined, represents a mechanistic or nonmechanistic immune correlate of protection (CoP). Based on other licensed flavivirus vaccines (Japanese encephalitis, Yellow fever) where correlates exist, one would assume that Nab titers are the best candidate for a CoP for dengue fever. However, preliminary assessments of clinical efficacy and immunogenicity data (plaque-reduction neutralization test) for a chimeric LAV dengue vaccine in a majority primed population did not support this assumption.^6^–^8^ Ongoing work in this field, including separate assessment of flavivirus-naïve and flavivirus-primed subjects, as well as modification of Nab assay methodology and utilization of cell-mediated immunity assays, will help to better understand the contribution of humoral and cellular responses to protection from disease.

A booster dose of DPIV, administered in the second year post primary vaccination, induced a strong anamnestic response against all four DENV serotypes (3- to 1,438-fold increase for DENV-1, 21- to 3,285-fold increase for DENV-2, 12- to 1,318-fold increase for DENV-3, and 35- to 8,016-fold increase for DENV-4), irrespective of vaccine formulation (alum- or AS01_E_-adjuvanted). Although long-term persistence data for the boost is lacking, the quantitative response to a booster dose is cause for optimism. Two subjects in the booster cohort, Subject A and E, had unexpected Nab titer kinetics (Table 3 and Figure 8) that could suggest flavivirus priming (Subject A, high plateau after dose 2) and exposure to a flavivirus while on study (Subject E, titer rise after M7). History regarding prior exposure, flavivirus vaccination, and travel were negative, however.

This report has several limitations. First, the study presented is a small phase 1 study with 20 subjects per treatment. Only descriptive statistics are provided and the study was not powered to demonstrate statistically significant differences between groups, neither with regard to immunogenicity nor to safety. Second, only 1 year of follow-up data on the entire cohort are reported here, and only humoral immunogenicity endpoints. Cellular immunity testing is ongoing and will be included in a subsequent report. Lastly, the booster dose was only administered to a few subjects and only two of the formulations were evaluated in an exploratory manner.

## Conclusion

This investigational DPIV had an acceptable safety profile in 100 volunteers 18–39 years of age. The 4 μg alum–adjuvanted as well as the AS03_B_- and AS01_E_-adjuvanted formulations induced high and balanced Nab responses to all four DENV serotypes 1 month after second vaccine dose in flavivirus-naïve healthy adult subjects. Nab titers waned during the first 6 months post-dose 2 and then stabilized at various levels depending on the formulation. A booster dose in the second year after primary vaccination induced strong anamnestic responses in two groups and in a limited number of participants, suggesting that B-cell memory lasts beyond the first year after primary vaccination. These FTiH study results support continuation of the clinical development of this dengue vaccine candidate.

## Figures and Tables

**Table 1:** Demographic characteristics of the study participants (total vaccinated cohort)

Characteristics	1 μg + alum	4 μg + alum	1 μg + AS01_E_	1 μg + AS03_B_	Placebo	Total
	*N* = 20	*N* = 20	*N* = 20	*N* = 20	*N* = 20	*N* = 100
Mean age, years (range)	28.2 (19–40)	26.1 (21–35)	28.0 (18–37)	28.4 (19–36)	28.3 (21–38)	27.8 (18–40)
Females, *n* (%)	11 (55%)	7 (35%)	7 (35%)	11 (55%)	7 (35%)	43 (43%)
African heritage/African American, *n* (%)	8 (40%)	13 (65%)	12 (60%)	9 (45%)	9 (45%)	51 (51%)
European heritage/White-Caucasian, *n* (%)	10 (50%)	4 (20%)	4 (20%)	8 (40%)	8 (40%)	34 (34%)

1 μg + alum indicates participants who received 1 μg/serotype/dose adjuvanted with alum; 4 μg + alum indicates participants who received 4 μg/serotype/dose adjuvanted with alum; 1 μg + AS01_E_ indicates participants who received 1 μg/serotype/dose adjuvanted with AS01_E_; 1 μg + AS03_B_ indicates participants who received 1 μg/serotype/dose adjuvanted with AS03_B_; *N*, number of participants; *n* (%), number and percentage of participants in a specific category.

**Table 2:** GMTs to each DENV serotype 28 days (ATP cohort for immunogenicity D56), 6 months (ATP cohort for immunogenicity M7), and 12 months (ATP cohort for immunogenicity M13) after the second vaccine dose

Serotype and group		Post-dose 2 (D56)		Post-dose 2 (M7)		Post-dose 2 (M13)	
		*N*	GMT (95% CI)	*N*	GMT (95% CI)	*N*	GMT (95% CI)
DENV-1	1 μg + alum	17	55 (34–88)	18	7 (5–10)	17	8 (5–12)
	4 μg + alum	16	179 (118–271)	17	14 (9–21)	17	13 (9–20)
	1 μg + AS01_E_	13	411 (215–785)	12	21 (10–47)	12	31 (10–101)
	1 μg + AS03_B_	16	526 (311–889)	16	33 (19–58)	14	25 (12–53)
	Placebo	16	5 (5–5)	17	5 (5–5)	17	5 (5–5)
DENV-2	1 μg + alum	17	34 (19–59)	18	6 (5–7)	17	7 (5–11)
	4 μg + alum	16	155 (91–265)	17	14 (8–23)	17	9 (6–15)
	1 μg + AS01_E_	13	484 (231–1,016)	12	20 (9–46)	12	27 (11–68)
	1 μg + AS03_B_	16	341 (175–663)	16	23 (12–41)	14	17 (9–34)
	Placebo	16	5 (5–5)	17	5 (5–5)	17	5 (5–5)
DENV-3	1 μg + alum	17	54 (35–82)	18	6 (5–7)	17	6 (5–7)
	4 μg + alum	16	191 (117–312)	17	13 (9–19)	17	9 (6–15)
	1 μg + AS01_E_	13	539 (275–1,059)	12	21 (10–46)	12	37 (15–91)
	1 μg + AS03_B_	16	468 (296–741)	16	29 (14–63)	14	24 (12–50)
	Placebo	16	5 (5–5)	17	5 (5–5)	17	5 (5–5)
DENV-4	1 μg + alum	17	20 (8–47)	18	6 (5–7)	17	5 (5–6)
	4 μg + alum	16	141 (58–345)	17	9 (5–16)	17	7 (4–13)
	1 μg + AS01_E_	13	307 (96–976)	12	7 (4–15)	12	9 (4–22)
	1 μg + AS03_B_	16	406 (192–859)	16	15 (7–35)	14	8 (5–14)
	Placebo	16	5 (5–5)	17	5 (5–5)	17	5 (5–5)

1 μg + alum indicates participants who received 1 μg/serotype/dose adjuvanted with alum; 4 μg + alum indicates participants who received 4 μg/serotype/dose adjuvanted with alum; 1 μg + AS01_E_ indicates participants who received 1 μg/serotype/dose adjuvanted with AS01_E_; 1 μg + AS03_B_ indicates participants who received 1 μg/serotype/dose adjuvanted with AS03_B_; ATP = according-to-protocol; GMTs = geometric mean antibody titers calculated on all participants (participants with a titer below the assay cutoff of 10 were attributed the arbitrary value of 5); Post-dose 2 (D56) = blood sampling 28 days post-dose 2 at Day 56; Post-dose 2 (M7) = blood sampling 6 months post-dose 2; Post-dose 2 (M13) = blood sampling 12 months post-dose 2; *N* = number of subjects with available data; 95% CI = 95% confidence interval.

**Table 3:** Neutralizing antibody titers to each DENV serotype before and 28 days after the booster dose for each participant included in the booster ATP cohort for immunogenicity

Group	Subject	DENV-1		DENV-2		DENV-3		DENV-4	
		Pre	Post	Pre	Post	Pre	Post	Pre	Post
1 μg + AS01_E_	A	1,370	4,781	172	3,659	387	4,494	186	6,477
	B	24	4,062	18	5,359	27	6,830	< 10	28,440
	C	19	1,690	37	3,864	67	2,172	< 10	3,557
	D	20	2,353	< 10	3,894	< 10	3,796	< 10	23,770
	E	444	6,235	157	4,234	291	19,790	567	16,970
	F	32	27,350	< 10	3,924	42	14,340	< 10	34,410
4 μg + alum	G	30	7,570	64	3,989	45	3,747	12	20,390
	H	< 10	710	< 10	153	< 10	246	< 10	528
	I	14	20,130	11	36,130	19	25,040	< 10	40,080

4 μg + alum indicates participants who received 4 μg/serotype/dose adjuvanted with alum; 1 μg + AS01_E_ indicates participants who received 1 μg/serotype/dose adjuvanted with AS01_E_; ATP = according-to-protocol; Pre = blood sampling prebooster; Post = blood sampling 28 days after booster; participants with a titer below the assay cutoff are indicated with a value as < 10.
